# Development of Standard Fuel Models in Boreal Forests of Northeast China through Calibration and Validation

**DOI:** 10.1371/journal.pone.0094043

**Published:** 2014-04-08

**Authors:** Longyan Cai, Hong S. He, Zhiwei Wu, Benard L. Lewis, Yu Liang

**Affiliations:** 1 State Key Laboratory of Forest and Soil Ecology, Institute of Applied Ecology, University of Chinese Academy of Sciences, Shenyang, People's Republic of China; 2 School of Natural Resources, University of Missouri-Columbia, Columbia, Missouri, United States of America; University of Missouri, United States of America

## Abstract

Understanding the fire prediction capabilities of fuel models is vital to forest fire management. Various fuel models have been developed in the Great Xing'an Mountains in Northeast China. However, the performances of these fuel models have not been tested for historical occurrences of wildfires. Consequently, the applicability of these models requires further investigation. Thus, this paper aims to develop standard fuel models. Seven vegetation types were combined into three fuel models according to potential fire behaviors which were clustered using Euclidean distance algorithms. Fuel model parameter sensitivity was analyzed by the Morris screening method. Results showed that the fuel model parameters 1-hour time-lag loading, dead heat content, live heat content, 1-hour time-lag SAV(Surface Area-to-Volume), live shrub SAV, and fuel bed depth have high sensitivity. Two main sensitive fuel parameters: 1-hour time-lag loading and fuel bed depth, were determined as adjustment parameters because of their high spatio-temporal variability. The FARSITE model was then used to test the fire prediction capabilities of the combined fuel models (uncalibrated fuel models). FARSITE was shown to yield an unrealistic prediction of the historical fire. However, the calibrated fuel models significantly improved the capabilities of the fuel models to predict the actual fire with an accuracy of 89%. Validation results also showed that the model can estimate the actual fires with an accuracy exceeding 56% by using the calibrated fuel models. Therefore, these fuel models can be efficiently used to calculate fire behaviors, which can be helpful in forest fire management.

## Introduction

Weather, terrain, and fuel are major factors influencing wildfire occurrence and behaviors [1]–[3], among which fuel is arguably the only factor human can mediate. The shape, size, loading, moisture content, and spatial configuration of forest fuels affect the ignition, intensity, spread, and effects of wildfire [4], [5]. Therefore, accurate information about the characteristics of fuels across a landscape is essential in fire management decision-making [6].

Fuel is complex spatially and temporally, changing with vegetation type, succession stage, and environments [7]–[9]. Due to the infinite combinations of vegetation type, sucession stage, and environment present in a landscape, it is impossible to characterize all possible combinations that affect fuel. Thus, generalizing fuels into finite number of fuel models has become a widely used approach to characterizing and mapping forest fuels across a landscape [10], [11]. A fuel model is defined as “an identifiable association of forest fuel components of distinctive species, form, size, arrangement, and continuity that will exhibit characteristic fire behavior under defined burning conditions” [12].

Fuel parameters (e.g., fuel load and fuelbed depth) have high complexity and variability in structure and distribution across a forest landscape [7]–[9]. Using a limited number of fuel models to cover such wide variations may be problematic in predicting fire behavior [13], [14]. Because fire behaviors vary with the fuel model parameters, analyses of fuel model parameter sensitivity can best understand the fuel variabilities or uncertainties, which subsequently can efficiently reduce parameter calibration workload [15]–[17] and improve fire behavior predictions accuracy.

The boreal forests of the Great Xing'an Mountains provide more timber and wood products than any other forested area in China, which store1.0–1.5 Pg C and contribution to approximately 24–31% of the total carbon storage in China [18]. The forests also encompass rather unique ecological and environmental settings in the region [19]. Historically, fires were primarily ignited by lightning in this area [20]. The historical fire regime was characterized by frequent and low intensity surface fires, with a fire return interval ranged from 30 years to 120 years [20]. However, current fires regimes in the region are characterized by infrequent and high intensity fires, with a fire return interval of more than 500 years, which has threatened the functions of the forest [21]. For example, on 6 May 1987, a catastrophic fire occurred in the northern slopes of Great Xing'an Mountains, burning a total area of 1.3×10^6^ ha, with disastrous impacts to forest composition and structure, and landscape pattern [22]. High fuel accumulation rate due to effective fire suppression since the 1950s is one of main reasons for fire intensity increasing in this situation. Thus, it is of great significance to study the fuel conditions in this region so as to carry out some effective management measures to reduce the potential losses caused by fires.

Numerous studies have been conducted on forest fuels and fire behaviors [23]–[27], with some having developed forest fuel models [23], [25], [28]. For example, Shan (2003) developed fuel models for the Great Xing'an Mountains region. These fuel models included *Larix gmelini* fuel model, *Betula platyphylla* fuel model, *Pinus pumila* fuel model, and *Betula platyphylla* fuel model, etc; Wu et al (2011) also developed four fuel models for the Huzhong Forest Bureau in the Great Xing'an Mountains region, including dense and heavily branched *Pinus pumila* shrublands fuel model, *Betula platyphylla* and *Populus davidiana* fuel model, coniferous forests fuel model with *Ledum palustre* and *Vaccinium uliginosum* shrub, and coniferous forests fuel model with *Rhododendron dauricum* shrub. However, fire behaviors (e.g., rate of spread and fire flame) of these fuel models only have been simulated using BEAHVE and have not been tested against actual fire behaviors. Whether these fuel models can reflect the spatial variations of fuel parameters well across the landscape is unknown. Therefore, it is of great importance to calibrate these fuel models in the future.

The overall objective of this study was to develop standard fuel models in the Great Xing'an mountains. Specifically, we intended to (1) determine the sensitivity of forest fuel model parameters using the Morris screening method; (2) calibrate parameters of fuel models; and (3) evaluate the efficiency of fuel models in fire behavior prediction with the FARSITE model.

## Materials and Methods

### Study area description

**Figure 1:**
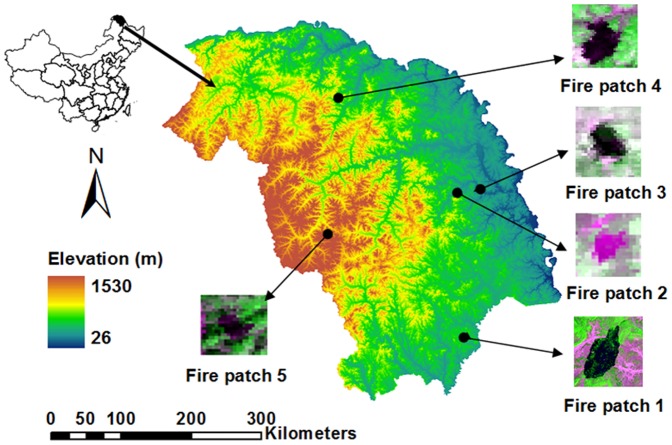
Study area with the five fire patches that were used to calibrate and validate fuel models developed in this study.

The study area is located on the northern and eastern slope of the Great Xing'an Mountains (121°12′∼127°00′ E, 50°10′∼53°33′ N) in northeastern China, and cover approximately 8.46×10^6^ ha (Fig. 1). This region has a long and severe continental monsoon climate. Average annual temperature is −6∼1°C. The coldest month is January with an average temperature of −38∼−28°C, and the hottest month is July, with an average temperature of 15∼20°C. Average annual precipitation is 240∼442 mm, 60% or more of which occurs between June and August.

The vegetation is representative of cool temperate coniferous forests in this region, forming the southern extension of the eastern Siberian boreal forests [19]. The overstory species include larch (*Larix gmelini*), pine (*Pinus sylvestris var. mongolica*), birch (*Betula platyphylla*), spruce (*Picea koraiensis*), willow (*Chosenia arbutifolia*), two species of aspen (*Populus davidiana* and *Populus suaveolens*), and a shrub species (*Pinus pumila*).

Historically, fires in this region were primarily ignited by lightning [20]. The fire regimes were characterized by frequent and low intensity surface fires, with a fire return interval ranged from 30 years to 120 years [20]. However, human activities (e.g., fire suppression and timber harvesting) have significantly changed natural fire regimes in this region [21], [29], [30]. For example, fire suppression in this region has been carried out for over a half century, which has lengthened the fire cycle with the fire return interval of longer than 500 years [22]. Currently, fires regimes are characterized by infrequent and more intensity fires and ignited by both human and lightning [30], [31].

### Overall study approaches

**Figure 2:**
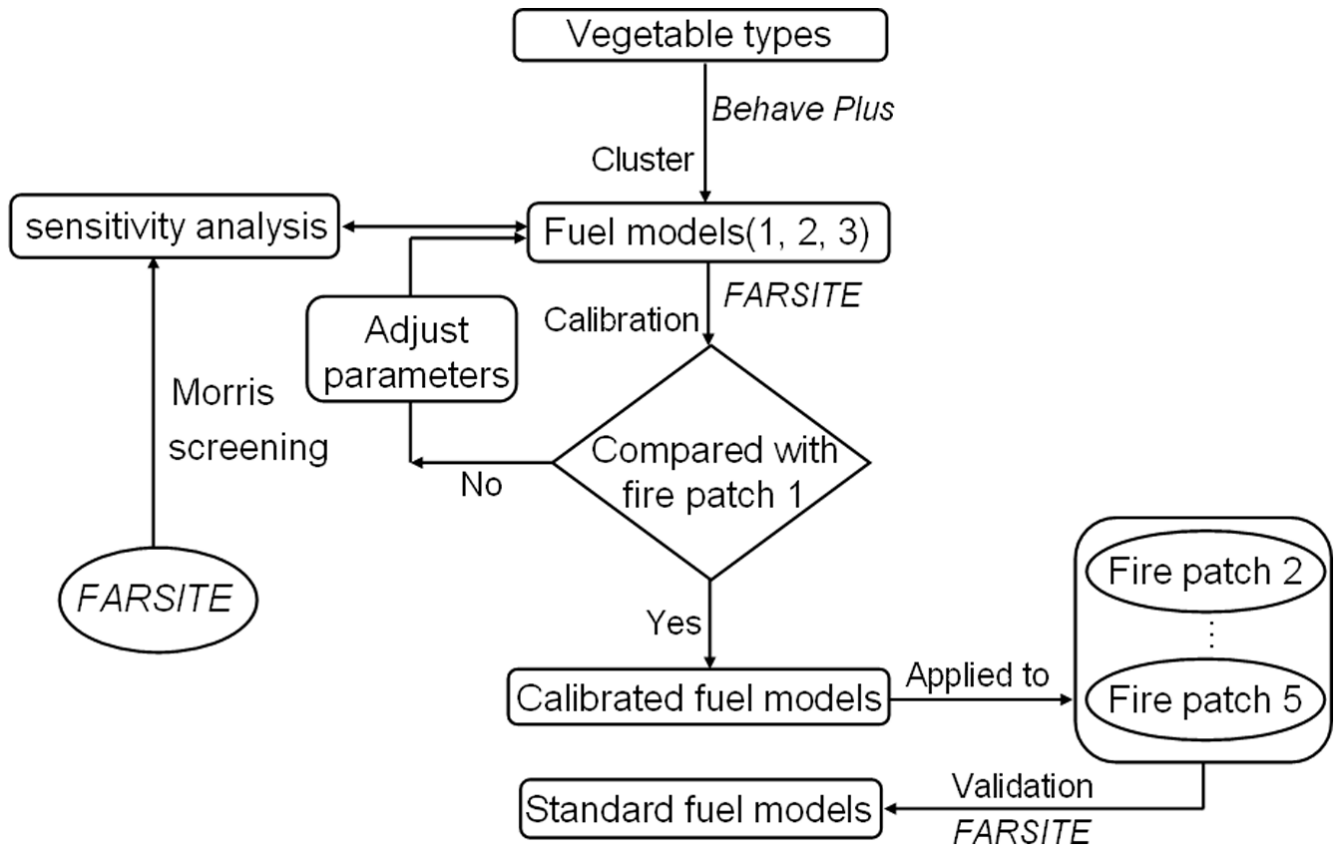
The overall study approaches.

To derive the fuel models, we first derived vegetation types such as meadow, shrub, swamp, deciduous broadleaf forest, deciduous coniferous forest, evergreen coniferous forest, and mixed coniferous and broadleaf forest from 1∶100 0000 vegetation map of our study area; these vegetation types were combined into a few number of fuel models based on the similarity of their potential fire behaviors simulated with BehavePlus 5.0 model [32]. We then conducted sensitivity analysis for parameters (e.g., fuel loading and fuelbed depth) of the three fuel models using the Morris screening method [33]. We used the default fuel model parameters to predict a series fires occurred in this region using FARSITE with the same fire weather conditions when the fire occurred. The reason to use FARSITE was because it predicted fire spread resulting in a fire patch that were comparable to actual fires. If discrepancies existed between the predicted and a set of actual fires, we iteratively adjusted fuel model parameters that had high sensitivity until acceptable matches were reached. Finally we applied the calibrated fuel models to another set of actual fires to gague how well these fuel models can predict actual fires (validation) (Fig. 2).

### Classification of forest fuel models

The BehavePlus 5.0 model was used to simulate fire behaviors of the seven vegetation types. Model inputs include fuel parameters (e.g., fuel loading and fuel bed depth), fuel moisture scenarios (Table 1), weather (midflame wind speed), and terrain conditions [34], [35]. In this study, fuel parameters were derived from Shan (2003).The wind conditions were simulated by setting 0–10 m/s for midflame wind speed (Shan 2003). All fire behavior simulations referred to zero slopes. The simulated potential fire behaviors included the rate of spread (m/s), heat per unit area (kJ/m^2^), fireline intensity (KW/m), and flame length (m).

**Table 1:** Fuel moisture content (%) scenarios [34] used for simulating fire behaviors of the seven vegetation types with BehavePlus model.

Fuel parameters	Very low	Low	Medium	High
1-hour time lag fuels	3	6	9	12
10-hour time lag fuels	4	7	10	13
100-hour time lag fuels	5	8	11	14
Live herbaceous fuels	30	60	90	120
Live shrub fuels	60	90	120	150

Hierarchical cluster analysis with relative Euclidean distances and Ward's method was used to identify forest fuel models by clustering the simulated potential fire behaviors of the seven vegetation types. If the potential fire behaviors were similar, the vegetation types were combined into one fuel model. After the clustering analysis, the parameters for a fuel model were assigned by the average values of vegetation types that were classified into the same cluster (Fig. 2). The cluster analysis process was performed with the SPSS 18.0 statistical software package [36], [37].

#### Historical fires

We selected five historical fire patches to conduct fuel model parameters sensitivity analysis, calibration, and validation (Table 2). The five historical fire patches were selected based on the following three considerations: (1) covering the three fuel models; (2) including different fire sizes; (3) representing the prevailing fire burning topographic conditions (e.g., aspects and elevations). In the fire simulations, we placed fire ignitions on the historical fire coordinates (Table 2).

**Table 2:** Descriptions of the five historical fire patches.

Fire patch	Fire date	Location	Fire size (ha)	Fuel models composition (area percent)	Topographic conditions		Utility	Source of fire patch	Burn duration
					Aspect	Elevation (m)			
1	May 26, 2000	125°38′10″ E, 50°39′00″ N	450	FM-1(8.8%) FM-2(40.5%) FM-3(50.7%)	Shady slope	460	Sensitivity analysis; Calibration	LANDSAT-ETM+	2:15 p.m.–11:25 p.m.
2	August 5,2005	125°35′15″ E, 50°59′09″ N	364	FM-1(31.6%) FM-2(56.8%) FM-3(11.6%)	Sunny slope	322	Validation	MOD09Q1	4:30 p.m.–3:03 a.m.
3	Apri 9, 2003	125°53′20″ E, 52°00′20″ N	580	FM-1(2.5%) FM-2(74.2%) FM-3(23.3%)	Shady slope	312	Validation	MOD09Q1	0:10 p.m.–10:30 p.m.
4	May 7, 2002	123°49′35″ E, 52°52′31″ N	1300	FM-1(55.3%) FM-2(22.6%) FM-3(22.1%)	Sunny slope	491	Validation	MOD09Q1	0:25 p.m.–10:35 p.m.
5	May 22, 2003	123°38′47″ E, 51°38′19″ N	234	FM-2(12.3%) FM-3(87.7%)	Sunny slope	986	Validation	MOD09Q1	4:23 p.m.–9:07 a.m.

#### FARSITE model

FARSITE was developed by USDA Forest Service [38] and was widely used to simulate fire behaviors [39]–[42]. FARSITE requires five raster-based themes (elevation, slope, aspect, fuel models, and canopy cover) and three crown fuel themes (stand height, crown base height, and crown bulk density), as well as meteorological files (wind, temperature, relative humidity and cloud cover) (Finney, 1998). More information on the FARSITE model can be obtained from Finney (1998).

The topography data (elevation, aspect, and slope) were derived from a 30 m Digital Elevation Models (DEM) from the U.S. Geological Survey (http://glovis.usgs.gov). Fuel model map was created (30 m spatial resolution) from the stand map in 2000s of the study area. The canopy fuel characteristics of canopy cover and tree height were derived from the stand map. The crown base height was derived from the field sample plots each with 20 m×20 m. The canopy bulk density was estimated based on crown biomass and volume equations from Chen et al. (2003) and Yu et al. (2010). The daily meteorological data were derived from the China Meteorological Data sharing Service System (http://cdc.cma.gov.cn) and weather online website (http://www.t7online.com). Initial fuel moisture content of fuels were derived from Wang et al. (2009).

Model parameters for the simulations were set to a time step of 30 min, perimeter of 30 m, and distance resolution of 20 m. During the simulations, the 24 hours conditioning period was used to adjust fuel moisture before the start of fire simulations. Fuel moistures were adjusted based on topography, weather and shading during the simulation [38]. We did not consider the effects of fire suppression. Water and roads were considered as nonfuel. The duration of each simulation for calibration and validation was derived from fire records of Heilongjiang province (Table 2).

#### Sensitivity analysis

We used the fire patch 1 to conduct the fuel model parameter sensitivity analysis (Fig. 1, Table 2). Six fire behaviors including burned area, spread perimeter, rate of spread, heat per unit area, fireline intensity, and flame length.were simulated with the FARSITE model. Based on the FARSITE simulations, the sensitivity of ten fuel model parameters were analyzed with the Morris screening method (Table 3)

**Table 3:** Parameters of the uncalibrated and calibrated fuel models.

Fuel model parameters	Uncalibrated (combined) fuel models			Calibrated fuel models		
	FM-1	FM-2	FM-3	FM-1	FM-2	FM-3
1-hour fuel loading(Mg/ha)/SAV(cm^−1^)	2.87/83.7	4.16/97.3	5.46/98.6	3.59/83.7	13.56/97.3	16.11/98.6
10-hour fuel loading(Mg/ha)/SAV(cm^−1^)	3.57/3.58	6.87/3.58	6.35/3.58	3.57/3.58	6.87/3.58	6.35/3.58
100-hour fuel loading(Mg/ha)/SAV(cm^−1^)	—	1.24/0.98	2.04/0.98	—	1.24/0.98	2.04/0.98
Live shrub(Mg/ha)/SAV(cm^−1^)	2.30/21.90	0.66/23.8	1.70/32.02	2.30/21.90	0.66/23.8	1.70/32.02
Fuel bed depth(cm)	36.45	18.39	29.46	43.15	52.17	83.57
Moisture of extinction (%)	52.20	40.19	36.62	52.20	40.19	36.62
Dead/live heat content (kJ/kg)	18942/20477	19847/20242	20820/21199	18942/20477	19847/20242	20820/21199

Note: Fuel model is a static fuel model and live meadow is included into the dead meadow in this study. SAV: Surface Area-to-Volume

The Morris screening method proposes a random One factor At a Time (OAT) design, in which only one input parameter
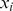
is adjusted between two successive runs of the model [33], [43]. The change induced onto the model outcome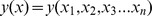 can then be unambiguously attributed to such a modification by means of an elementary effect
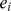
defined by [33]:
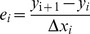
(1)


Where 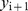 is the new outcome, 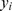
the previous one, 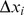 is the variation in the parameter 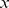.

In the revised Morris screening method, the influence of the change of the factor is indicated through the formula: 
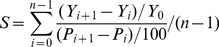
(2)


Where 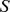 is sensitive identification index; 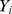 is model outcome for time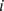; 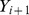 is the model outcome for time (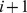); 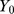 is the initial value of the model output before calibration; 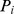 is the change percentages of parameter before and after calibration for time 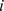; 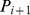 the change percentages of parameter before and after calibration for time (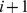); 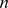 is the number of predictions. The simulation duration was 24 h. In this process these parameters were assumed to have a uniform distribution and to be independent of each other. Some very sensitive parameters were selected for calibration and validation to find the most reasonable values.

Sensitive Identification Indexes (SII) of fuel model parameters were ranked into four classes (Table 4) [44].

**Table 4:** Criterion used for ranking fuel model parameter sensitivity.

Class	Index	Sensitivity
I	|SII|≥1.00	Very high
II	0.20≤|SII|<1.00	High
III	0.05≤|SII|<0.20	Medium
IV	0.00≤|SII|<0.05	Negligible

Note: SII: Sensitive Identification Index.

### Calibration and validation of fuel model parameters

Historical fire patch 1 was also used to calibrate the parameters of the combined three fuel models (Table 3, uncalibrated fuel model). If the fuel models can predict the historical fire well, the fuel models were deemed valid; otherwise, we varied high sensitivity fuel model parameters within the possible range of variability to determine the fuel models that best predicted fire behaviors (Fig. 1). The criterion for the best prediction was the simulated fire size matched 90% the historical fire size. Four patches (Table 2) (Fig. 1, Fire patches 2 to 5) were used to validate the calibrated fuel models.

In the fuel model calibration and validation processes, the Fire Prediction Accuracy (FPA) was defined as:

FPA (%) = (Simulated fire size ∩ Historical fire size)/Historical fire size.

The FPA indicates whether the simulated fire close to the historical fire size.

## Results

### Forest fuel models description

**Figure 3:**
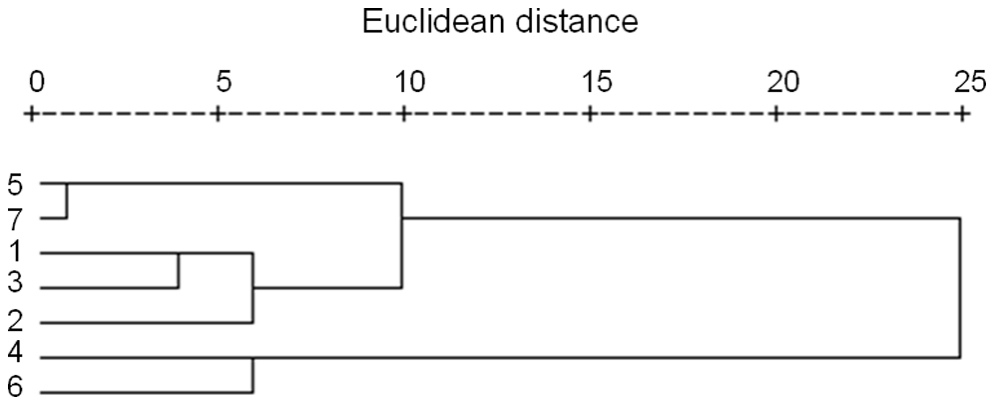
Fire behaviors clustering diagram of the seven vegetation types using SPSS 18.0. The clustered fire behaviours included rate of spread, heat per unit area, fireline intensity, and flame length.

**Figure 4:**
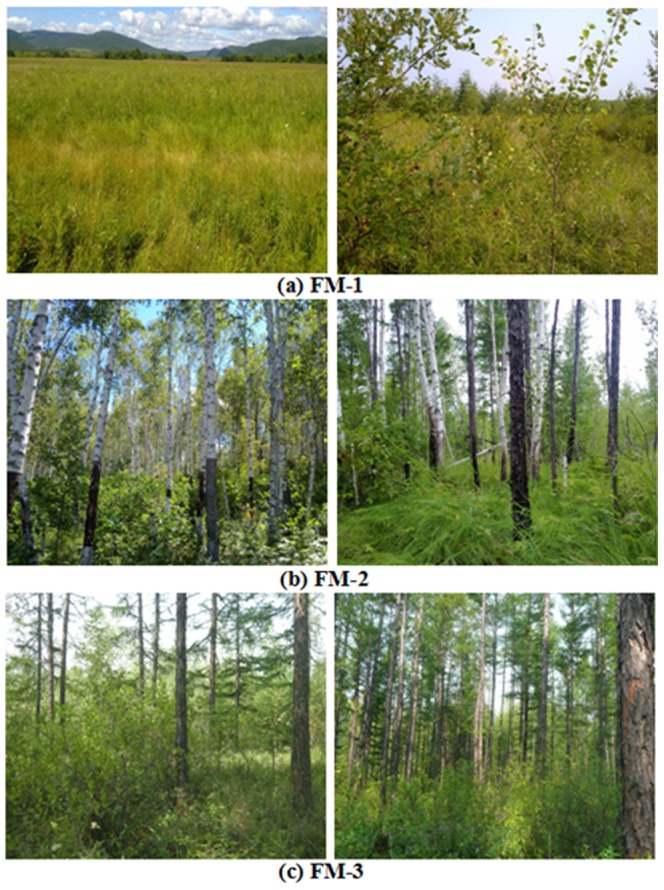
Example photos of fuel types.

We derived seven vegetation types from the 1∶1,000,000 vegetation map, which were combined into three fuel models according to their potential fire behaviors (Fig. 3) (Note: 1: Meadow; 2: Shrub; 3: Swamp; 4: Evergreen coniferous forest; 5: Deciduous broadleaf forest; 6: Deciduous coniferous forest; 7: Mixed coniferous and broad-leaf forest). Fuel model 1 (FM-1) is a non-forest fuel model (including shrub, meadow, and swamp) (Fig. 4a), which covers about 26.3% of the study area. Grasses are well developed in these three vegetation types with the average high of 60∼90 cm. The fuel model 1 burns surface fires with higher spread rate. Fuel model 2 (FM-2) is dominated by broadleaf forests (including deciduous broadleaf forests, and mixed coniferous and broadleaf forests), which covers about 29.7% of the study area (Fig. 4b). This fuel model had the lowest fire spread rate. Fuel model 3 (FM-3) includes deciduous coniferous forests and evergreen coniferous forests (Fig. 4c), which covers about 39.4% of the study area. This fuel model possesses higher fuel loading (dead fuel loading) (Table 3) and had the highest fire spread rate.

### Fuel model parameter sensitivity

**Figure 5:**
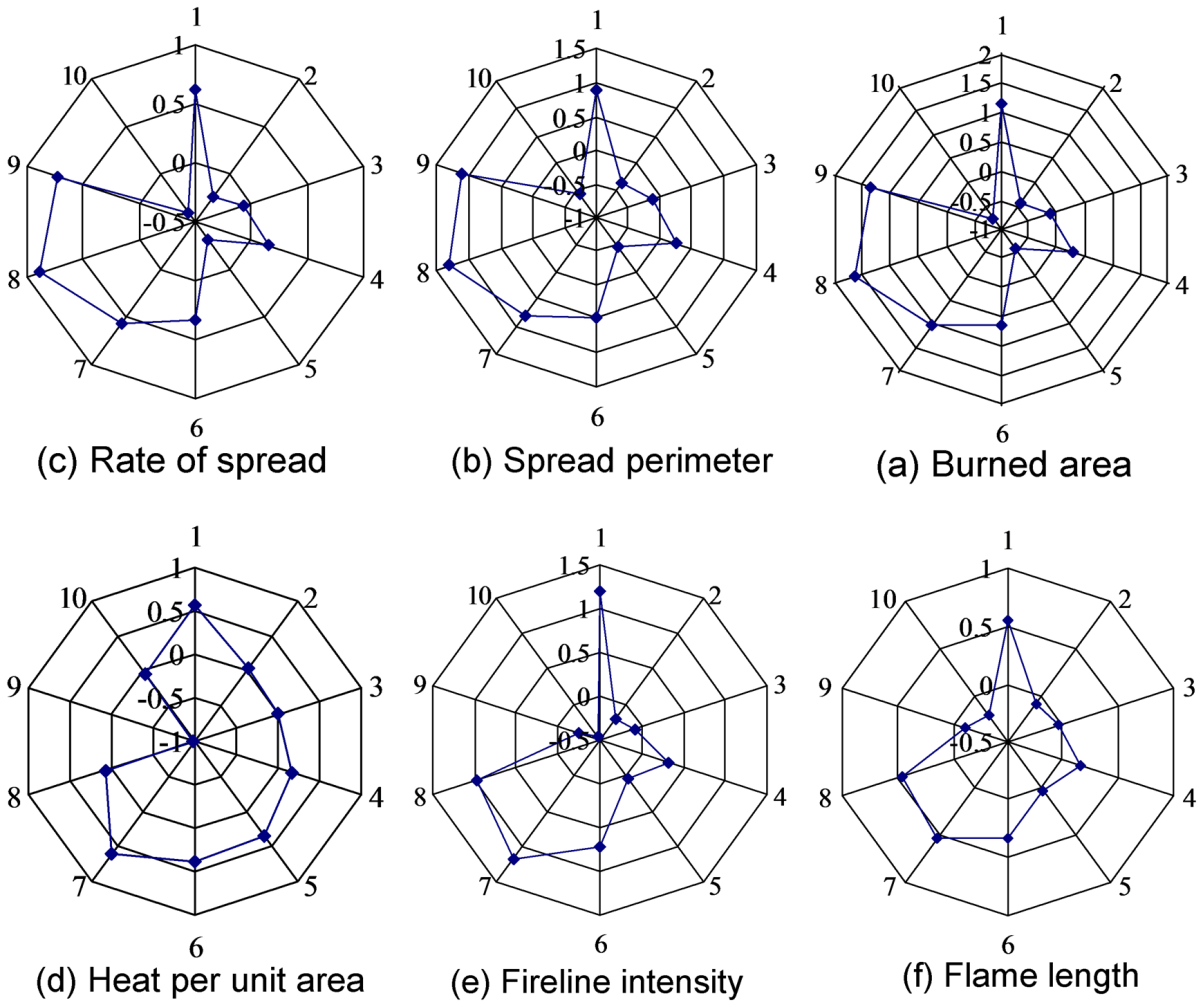
Scatter diagram of the fuel model parameter sensitivity.

Fuel model parameters of 1-hour time-lag loading, dead heat content, live heat content, 1-hour time-lag SAV(Surface Area-to-Volume), live shrub SAV, and fuel bed depth had high sensitivity (Fig. 5) (Note: 1: 1–hour time–lag loading; 2: 10–hour time–lag loading; 3: 100–hour time–lag loading; 4: Moisture of extinction of dead fuel; 5: Live shrub loading; 6: Live heat content; 7: Dead heat content; 8: Fuel bed depth; 9∶1–hour time–lag SAV(Surface Area-to-Volume); and 10: Live shrub SAV. The absolute value of sensitive identification index from -1 to 2 represent the degree of sensitivity.). Of these fuel model parameters, 1-hour time-lag loading and fuel bed depth have high spatio-temporal variability. Therefore, 1-hour time-lag loading and fuel bed depth were selected as the adjustment parameters for calibration. The range of 1-hour time-lag loading and fuel bed depth varied from 0 to 20 Mg/ha and fuel bed depth varied from 0 to 2 m [23].

### Calibration and validation of fuel model parameters

**Figure 6:**
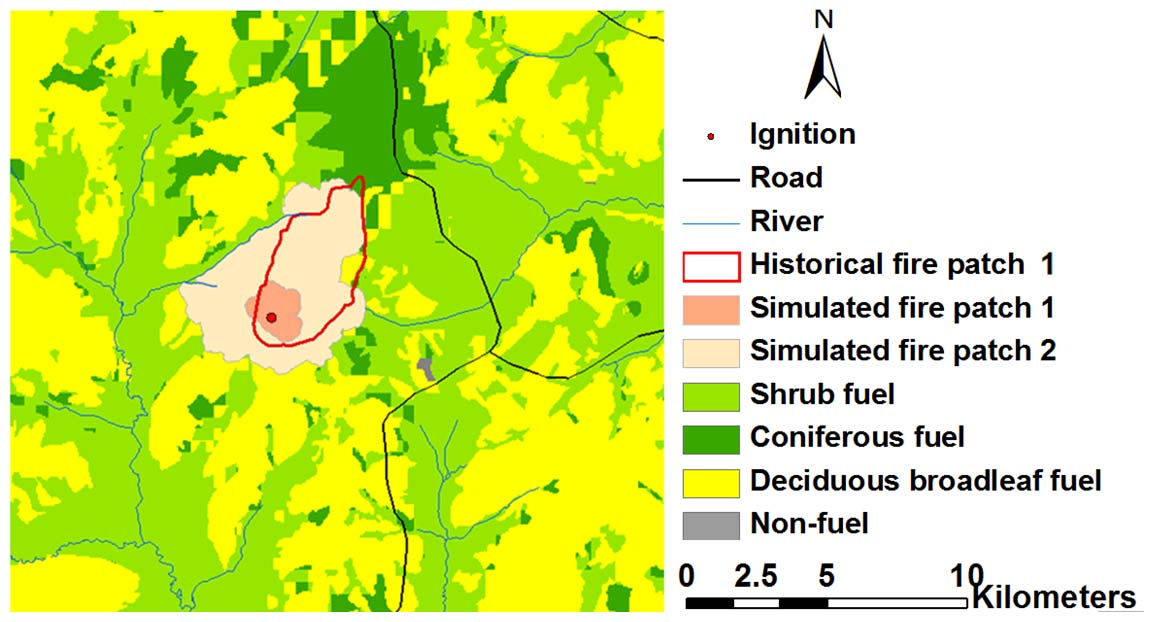
Calibration results of fire size between uncalibrated and calibrated fuel models.

The calibrated fuel model (Table 3) can be determined as the set of standard fuel models of the Great Xing'an Mountains.The prediction accuracy of fire patch 1 using the uncalibrated fuel models was only 17% (Fig. 6) (Note: Simulated fire patch 1 was derived using the uncalibrated fuel models. Simulated fire patch 2 was derived using the calibarted fuel models.). This indicates that the uncalibrated fuel models in FARSITE simulation produced an unrealistic prediction of fire. In contrast, FARSITE model estimated the actual fire size (fire patch 1) with an accuracy of 89% using the calibrated fuel models (Fig. 6).

**Figure 7:**
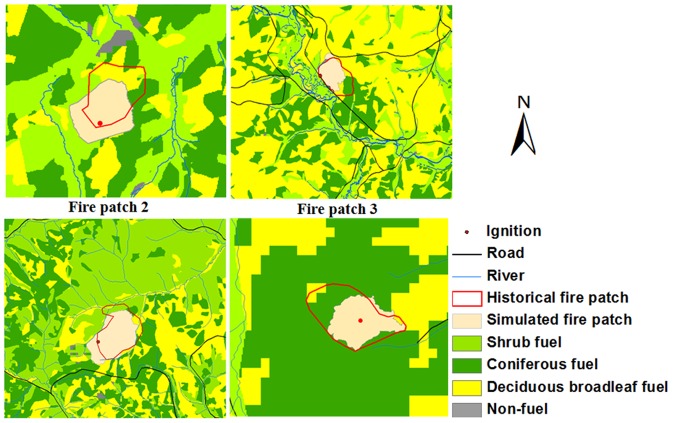
Validation results of calibrated fuel models in simulating historical fires.

The validation accuracy of fire size (fire patches 2–5) ranged from 56% to 76% (Fig. 7) with average of 64%. The highest accuracy was observed in fire patch 4 (76%), followed by fire patch 3 (63%) and fire patch 5 (61%), and finally lowest was fire patch 2 (56%).

### Comparing with fuel models in other regions

We compared the fuel model parameters and their fire behaviors with those in the United States and the Mediterranean regions. Standard shrub fuel models developed in the United States include FM4 (chaparral), SH2 (moderate load, dry climate shrub), SH3(moderate load, humid climate shrub), SH5 (high load, dry climate shrub), SH6 (low load, humid climate shrub), SH7 (high load, dry climate shrub), and SH8 (high load, humid climate shrub) [34], [35]. The CM28, a custom shrub fuel model was developed for maquis vegetation in the Mediterranean area [13]. TL2 (Low load broadleaf litter), TL6 (high load broadleaf litter), and TL9 (very high load broadleaf litter) are standard broadleaf fuel models, whereas TL1(low load, compact conifer litter), TL3 (moderate load confier litter), and TL5 (high load conifer litter) are standard coniferous fuel models developed in the United States [34]. Weather and fuel moisture contents used for fire behavior predictions using Behave Plus model were given (Table 5).

**Table 5:** Weather and fuel moisture contents used for simulating fire behaviors of the calibrated fuel models (The parameters represented the prevailing fire weather conditions of the historical fires).

Fuel moisture and weather	Values
1-hour moisture content (%)	12
10-hour moisture content (%)	13
100-hour moisture content (%)	14
Live herbaceous fuel moisture content (%)	170
Live shrub fuel moisture content (%)	170
Wind speed (km/h)	15

Our standard fuel model 1 (FM-1, Table 3) exhibited similar dead fuel load to SH3, live fuel load to SH6, and fuel bed depth to SH2. However, the SAV (Surface Area-to-Volume) of 1-hour time-lag fuel and live shrub as well as the live/dead heat contents were different from the shrub fuel models developed in other regions. The standard fuel model 2 (FM-2, Table 3) and standard fuel model 3 (FM-3, Table 3) have fuel model parameters that are different from those of these standard broadleaf and coniferous fuel models.

Fire prediction capabilities of standard fuel model 1 (FM-1, Table 3) vary from those of the shrub fuel models in other regions (Table 6). However, the standard fuel model 1 in this study was found to be most similar to FM4. For standard fuel model 2 (FM-2, Table 3), fire behaviors are higher compared with those of other standard broadleaf fuel models except for the heat per unit (lower compared with that of TL9) (Table. 6). For standard fuel model 3 (FM-3, Table 3), fire behavior are higher than those of other standard coniferous fuel models (Table 6).

**Table 6:** Comparing fire behaviors with that of fuel models in other regions.

Fire behaviors	Shrub fuel models									Deciduous broadleaf fuel models				Coniferous fuel models		
	FM4	SH2	SH3	SH5	SH6	SH7	SH8	CM28	FM-1	TL2	TL6	TL9	FM-2	TL1	TL5	FM-3
ROS(m/min)	18	0.7	1.4	9	8	5.4	3.2	5.2	28.6	0.4	3.5	4.7	13.7	0.2	2.5	132.8
HPA(KJ/m2)	13358	3030	4672	6775	15210	8580	8371	4794	7807	1445	4377	9847	5163	1014	3479	21237
FLI(KW/m)	4000	35	106	1020	2017	778	449	419	3715	11	253	774	1175	3	144	46995
FML(m)	3.5	0.4	0.7	1.9	2.6	1.7	1.3	1.2	3.4	0.2	1	1.7	2	0.1	0.8	10.9

Note: ROS: Rate of Spread; HPA: heat per unit area; FLI: fireline intensity; FML: flame length.

## Discussion

Forest fuels have high spatial complexity and variability in structure and distribution across a landscape [7]–[9]. Therefore, fuels are difficult to inventory, classify and describe [4], especially in a highly heterogeneous forest landscape. Generally, sampling location, sampling range, sample quantity, and professionalism of surveyors can significantly affect fuel model parameter representativeness [45]–[48]. In order to reduce uncertainty of fuel model parameters caused by limitations of measures, one needs to pay more attention to the highly variable parameters that have high uncertainty in fire simulation.

The fuel model parameter sensitivity analysis is particularly useful in identifying the uncertainty sources of fire behavior prediction [49]. Our results of sensitivity analysis showed that 1-hour time-lag loading, 1-hour time-lag SAV (Surface Area-to-Volume), fuel bed depth, dead/live heat content, and live shrub SAV are sensitive for fire behaviors prediction. Special attentions in field sampling should be paid to reduce uncertainties of these parameters. Some studies have also showed that 1-hour time-lag loading and fuel bed depth were the two main parameters affecting fire behaviors [50], [51]. For example, Sparks et al. (2002) found that fireline intensity increased significantly as 1-hour time lag fuels increased in restored shortleaf pine–grassland communities.

The parameters of the fuel models in this study were static. However, fuel model parameters change with natural and human disturbances [52], [53]. For example, fire suppression have significantly increased the fuel load of many forest ecosystems [22], [54]. Moreover, several studies suggested fuel parameters change with climate conditions and vegetation succession [55]. The variation of fuel model parameters may cause some uncertainty in fire predition. In this study, FARSITE model yielded an unrealistic prediction of the historical fire using the uncalibrated fuel models (Fig. 6). This was because the fire (fire patch 1) used for parameter calibration occurred in 2000, while fuel model parameters were measured in 1990s [23]. Fuel model parameters (e.g., fuel load and fuelbed depth) may significantly change after about 10 years [56], which may lead to low fire prediction accuracy [13], [14]. The validation of the prediction accuracies also declined over time, which can be attributed to the increasing of fuel load and change of fuel structure, particularly in 1 hour time-lag loading and fuel bed depth (Table 3, and 6). Weather and topography are also two source of uncertainty in predicting fire behavior [13], [57]. For example, FARSITE model does not account for topographic variations that affect wind exposures to surface fires. Also, lee-side topographic sheltering can undoubtedly cause errors for spread rate calculations[58].Therefore, to reduce the uncertainty of fuel model parameters, fuel models are need to be calibrated and validated with more fires.

Our calibration and validation results indicated that the calibrated fuel models could predict historical fires well (Fig. 7). The calibrated fuel models can quantify surface fuel characteristics in this region, which is consistent with Hu (2005). The overpredictions were also observed in the fire prediction (Fig. 7), which can be explained by several reasons. For example, fire suppression was not considered in this study. The assumptions and limitations of the FARSITE model also significantly affect fire prediction accuracy [38]. Some previous studies suggested that the FARSITE simulations generally overpredict fire spead rate for all fuel models implemented with the Rothermel spread equation [59] and ascribed the cause to the relation of wind speed to elliptical dimensions [38].

We tested the efficiencies of fuel models in predicting fire size and other fire behaviors such as fire intensity and rate of spread were not considered in this study. Some studies have also used fire perimeters and fire size to calibrate fuel model [39], [60]. Fire perimeters and fire size are easy to obtain from field work or remote sensing, which can greatly reduce workload of obtaining such fire behaviors as fireline intensity and rate of spread. However, it should be noted that only using fire size to calibrate fuel models may still have uncertainty, because fire size is not a linear results of the rate of spread [61], [62]. Ideally, more fire behavior parameters for fuel model calibration and validation could further improve the reliability of fuel models identified in our study. However, such real time fire behavior data were lacking. Thus, one way to deal with this uncertainty is to use more fires to validate the fuel models [63], [64].

The fuel models developed in this study (Table 3) are different from those of other regions [34], [35]. This can mainly be explained by long-term fire suppression in this region. Fire suppression has been carried out for over a half century in China [22]. The Chinese government has invested greatly in both funding and manpower, including the army, forestry policemen, and local residents for fire control [65], which had signicantly increased the fuel load and subsequently caused different fire behaviors from that of fuel models in other regions (Table 6). Some studies have showed that fire behaviors were significantly affected by fuel model parameters [14], [66]. Our sensitivity analysis results also showed that fuel model parameters of 1-hour time-lag loading, dead heat content, live heat content, 1-hour time-lag SAV (Surface Area-to-Volume), live shrub SAV, and fuel bed depth had high sensitivity. However, in fire behavior modelling heat content is assigned a value around 18000–19000 kJ/kg and it is essentially assumed constant [34], [35]. Because heat content of fuels varies within a restricted range that is about plus or minus 10–20% of a nominal value. Generally, there is also same SAV (Surface Area-to-Volume) for a type of fuel model [34], [35]. For example, SH6 fuel model (low load, humid climate shrub), SH7 fuel model (high load, dry climate shrub), and SH8 fuel model (high load, humid climate shrub) developed in the United States have the same heat content and SAV (Surface Area-to-Volume). Therefore, of fuel model parameters, 1-hour time-lag loading and fuel bed depth, are the main sources of discrepancies between our fuel models and that of other regions. In addition, using different model-based outputs (e.g., FARSITE and BEHAVE) to calibrate fuel model may another reason for the differences between our fuel models and that of other regions. In this study, we used historical fires to calibrate fuel models and used FARSITE model to predict fire behaviors. Because BEHAVE is a non-spatial model, while FARSITE is a spatially explicit model that can predict fire behaviors across a landscape [13], [57], [67].

## Conclusions

We were able to derive three fuel models for northeastern China through calibrating the sensitive fuel model parameters and testing the calibrated fuel model parameters against historical fires. The sensitivity analysis results showed that fuel model parameters of 1-hour time-lag loading, dead heat content, live heat content, 1-hour time-lag SAV(Surface Area-to-Volume), live shrub SAV, and fuel bed depth often had high sensitivity. Of the sensitive fuel model parameters in fire behavior prediction,1-hour time–lag loading and fuel bed depth, were determined as adjustment parameters due to their have high spatio-temporal variability. The results showed that calibrated fuel models have significantly improved the capabilities of the fuel models to predict the actual fire with an accuracy of 89%. The prediction accuracy of the validation studies exceeded 56%. Therefore, developing standard fuel models through sensitivity analyses is practical because it improves the representativeness of parameters in our study area. The fuel models developed in this study can be useful for forest management and fire prediction.
